# Prevalence, Risk Factors, and Co-Occurrence of *Candida* Species and Bacterial Vaginosis Among Women in the Eastern Cape, South Africa: A Cross-Sectional Study

**DOI:** 10.3390/microorganisms14081691

**Published:** 2026-08-01

**Authors:** Samkelisiwe Beje, Hannibal T. Musarurwa, Zizipho Z. A. Mbulawa

**Affiliations:** 1 School of Pathology, iYunivesithi Walter Sisulu, Mthatha 5100, South Africa; 219260613@mywsu.ac.za; 2 School of Biomedical Sciences, iYunivesithi Walter Sisulu, Mthatha 5100, South Africa; hmusarurwa@wsu.ac.za

**Keywords:** *Candida* species, women, bacterial vaginosis, vaginal dysbiosis, *Lactobacillus* species

## Abstract

The cervicovaginal microflora plays a vital role in maintaining vaginal health, and disturbances can predispose women to opportunistic infections and sexually transmitted infections (STIs). This study examined the prevalence and associated factors of *Candida* species, bacterial vaginosis (BV), and their co-occurrence among women in the Eastern Cape province of South Africa. A cross-sectional design was employed, enrolling 325 women aged 18–60 years from a health facility in the Eastern Cape, South Africa. *Candida* species were identified in cervical specimens using a Seegene Allplex™ Candidiasis Assay. BV status and BV-associated bacteria were identified via Seegene Allplex™ Bacterial Vaginosis Plus assays. Overall, 64.51% of participants were HIV-positive, 15.74% tested positive for *Candida* species, 52.85% tested positive for BV, and 7.10% were positive for BV–*Candida* species co-occurrence. The highest prevalence of *Candida* species (23.60%; R^2^ = 0.913; *p* = 0.015), BV (85.39%; R^2^ = 0.931; *p* = 0.003), and *Candida*–BV co-occurrence (7.10%; R^2^ = 0.903, *p* = 0.041) was observed in the 18–29 age group and declined significantly with age. Women with four to six lifetime sexual partners had higher odds of testing positive for *Candida* species (AOR: 2.42, 95% CI: 1.21–4.82, *p* = 0.012). Currently presenting with STI symptoms (AOR: 4.66; 95% CI: 1.29–16.87; *p* = 0.019), increased (four to six) lifetime sexual partners (AOR: 5.58; 95% CI: 1.16–26.82; *p* = 0.032), and being HPV-positive (AOR: 7.28; 95% CI: 1.17–45.26; *p* = 0.033) were independent predictors of *Candida*–BV co-occurrences. In this current study, having multiple lifetime sexual partners was independently associated with *Candida* species positivity, while current STI symptoms, HPV infection, and having four to six lifetime sexual partners significantly increased the likelihood of *Candida*–BV co-occurrence, highlighting the role of sexual behaviour in the vaginal microbial profile. This underscores the need for continued surveillance and longitudinal investigation. Longitudinal studies to further investigate these associations are warranted.

## 1. Introduction

As a component of the larger microbiome of the female genital tract [1], the fungal community is maintained in balance by host immunity and the dominant lactic acid-producing bacteria to sustain homeostatic health [2]. Changes in the vaginal microbial ecosystem, commonly referred to as dysbiosis, may lead to yeast infections or vulvovaginal candidiasis (VVC) [2]. The proliferation of *Gardnerella vaginalis*, elevated vaginal pH, and reduction in protective *Lactobacillus* species are common markers of vaginal dysbiosis [3,4,5]. This often leads to the manifestation of bacterial vaginosis (BV), which not only causes localised discomfort but also opportunistic infections and increased chances of sexually transmitted infections (STIs) [6].

The development of VVC is often mediated by *Candida albicans*, which dominates the vaginal mycobiome due to its morphological plasticity, enabling the transition from commensal blastoconidia to invasive hyphae that express virulence factors such as candidalysin [2]. This transformation, along with immune surveillance by IL-17-producing γ/δ T cells and hyphae-triggered neutrophil recruitment via c-Fos/MAPK signalling, is the leading cause of vaginal inflammation and its clinical signs [1,2,7]. This effect is often accompanied by a lack of *Lactobacillus* spp., which confer their protective effects by producing lactic acid, hydrogen peroxide, and bacteriocins that suppress pathogenic growth [1,2,6].

Although BV, VVC, and associated STIs are sometimes not life-threatening, they carry a psychosocial burden and can be physically debilitating. They are often accompanied by distressing symptoms such as vaginal pruritus (itching), burning, and dyspareunia, along with observable clinical signs such as vaginal erythema and oedema [7]. Beyond discomfort, the resulting inflammatory environment and microbial imbalance can negatively impact reproductive health and conception and increase the risk of human immunodeficiency virus (HIV), other STIs, and their associated outcomes [8]. Despite this, there is limited epidemiological data among women in the Eastern Cape province on vaginal *Candida* species, BV, and their association with susceptibility to STIs. Therefore, this study examined the prevalence and associated factors of *Candida* species, bacterial vaginosis (BV), and their co-occurrence among women in the Eastern Cape province of South Africa.

## 2. Materials and Methods

### 2.1. Study Setting and Population

This is a cross-sectional study that enrolled women aged 18–60 years who attended the Gateway Clinic, a public primary health facility in the King Sabata Dalindyebo (KSD) Local Municipality in the Eastern Cape Province, South Africa. The clinic serves a diverse population across KSD, including rural, peri-urban, and informal settlement areas. Women who visited the clinic for any reason, such as accompanying someone, picking up medication, or seeking medical assistance, were invited to participate in the study. By including women from various locations and diverse healthcare needs, this study aimed to obtain a broad representation of the population within the KSD Municipality.

### 2.2. Recruitment and Enrolment of Study Participants

The study population was previously reported by Mbulawa et al. (2024) [9]. Study participants were recruited and enrolled via convenience sampling from June to August 2023. Recruitment occurred at a public primary health facility, the Gateway Clinic, where individuals visiting for any reason were invited to participate. Eligible participants were women aged 18–60 years old who were currently or previously sexually active, not pregnant, and not menstruating during the period of enrolment. Written consent was obtained from all participants; those unable to write signed the consent form using a thumbprint. All participants willingly agreed to provide biological specimens and respond to the questionnaire. Participation in the study was voluntary; participants were not pressured, coerced, or obligated to participate.

### 2.3. Sample Size Determinations

The sample size was estimated using the following formula as described by Naing et al. [10]:

$$s=\frac{Z^{2}\times P(1-P)}{m^{2}}$$

where s = sample size; z = z statistic for a level of confidence (1.96 for 95% confidence level); P = expected prevalence or proportion of 27.58%, estimated based on the most prevalent *Candida* spp. reported by Beje and Mbulawa et al., 2026 [11]; and m = margin of error (estimated deviation of 6% from the actual population). An additional 15% was added for non-respondents, and the total number was rounded up to the nearest hundred. The required sample size was 245 and was rounded up to 300 to ensure adequate statistical power. Therefore, a minimum sample size of 300 participants was deemed sufficient to estimate the prevalence, distribution, and diversity of *Candida* and BV-causing and BV-protective species, as well as associated factors.

### 2.4. Data Collection

#### 2.4.1. Questionnaire

Consented participants were interviewed privately by the investigator. A semi-structured questionnaire was used to collect data on demographics, reproductive history, sexual behaviours, smoking habits, alcohol consumption, hygienic routines, and the type of underwear material commonly used. Participants self-completed the questionnaires, while the investigator read each question aloud and explained it. However, in a few cases where participants could not complete the questionnaires on their own, participants responded verbally while the investigator completed the questionnaire on their behalf.

Participants were also asked to report their HIV status. Those who had either a negative or an unknown HIV status were offered the HIV rapid test. Prior to HIV testing, pre- and post-HIV counselling was conducted by a qualified nurse or HIV lay counsellor. In accordance with the HIV recommendations of the South African Department of Health, all individuals who tested positive for HIV received the necessary follow-up. It is important to mention that conducting HIV testing was a prerequisite to participating in the study. Each participant was assigned a unique study number that linked the specimen and questionnaire.

#### 2.4.2. Specimens and Laboratory Investigation

Cervical samples were collected by a qualified nurse, stored, and transported in a Digene transport medium (Qiagen, Inc., Gaithersburg, MD, USA) to the laboratory within three hours of collection. Thereafter, they were stored at −20 °C in the laboratory until nucleic acid extraction was performed. It is important to note that while the nurse was collecting specimens, syndromic management was performed to assess for STIs. An automated Seegene NIMBUS (Seegene Inc., Seoul, South Korea) extraction system, in conjunction with the Universal STARMag extraction kit (Seegene Inc., Seoul, Republic of Korea), was used to extract total nucleic acids from cervical samples according to the manufacturer’s instructions. To identify *Candida* species, the extracted nucleic acid was processed using the Allplex™ Candidiasis Assay (Seegene Inc., Seoul, Republic of Korea), a multiplex real-time polymerase chain reaction (PCR) assay, as per the manufacturer’s instructions. The Allplex™ Candidiasis Assay detects seven different *Candida* species known to cause candidiasis: *Candida albicans*, *Candida dubliniensis*, *Candida glabrata*, *Candida krusei*, *Candida lusitaniae*, *Candida parapsilosis*, and *Candida tropicalis*, all in a single analysis. Bacterial vaginosis was estimated using the Seegene Allplex™ Bacterial Vaginosis Plus Assay (Seegene Inc., Seoul, Republic of Korea), a real-time PCR assay that detects *Lactobacillus* species (*L. crispatus*, *L. gasseri* or *L. jensenii*), *Gardnerella vaginalis*, *Atopobium vaginae*, *Bacteroides fragilis*, *Mobiluncus* spp., *Megasphaera* type 1, and BV-associated bacteria 2 (BVAB2). Additionally, both the Seegene Allplex™ Candidiasis Assay and Allplex™ Bacterial Vaginosis Plus Assay target the β-globulin gene as an internal control to monitor sample adequacy, nucleic acid extraction, and amplification. Amplification was performed on a CFX96 real-time thermocycler (Bio-Rad, Hercules, CA, USA) according to the manufacturer’s instructions. Seegene Viewer Software v3.31.000.006 (Seegene Inc., Seoul, Republic of Korea) was used to view and interpret the generated data. Then, BV was identified by quantitative detection of *Lactobacillus* spp. (*L. crispatus*, *L. gasseri* or *L. jensenii*), *Gardnerella vaginalis*, *Atopobium vaginae*, *Bacteroides fragilis*, *Mobiluncus* spp., *Megasphaera* type 1, and BVAB2. BV-positive, BV-intermediate, and two sub-classifications of BV-negative status were assigned. The BV-negative status was distinguished as having an appreciable quantity of *Lactobacillus* species (quantitative thresholds ≥ 3.74 log) or lacking appreciable quantity (quantitative thresholds < 3.74 log) to identify individuals in a state of ‘pre-dysbiosis’ or age-related microbial depletion and despite lacking active infection markers.

### 2.5. Statistical Analysis

All questionnaire and laboratory investigation variables were captured and coded in Microsoft Excel, then transferred to GraphPad Prism 8.0 and IBM SPSS Statistics version 30 for in-depth analysis. Data were presented using tables, median, and interquartile range (IQR). Depending on the expected frequency, two categorical variables were compared for association using the Chi-squared test or Fisher’s exact test. The Fisher’s exact test was used if 20% or more of the cells had expected frequencies of less than 5, or if any single cell had an expected frequency of 0; otherwise, the Chi-squared test was used. Spearman’s rank correlation (R^2^) was used to analyse age trends. Bivariate and multivariable logistic regression models were used to investigate factors associated with *Candida* species and its co-occurrence with BV. Multivariable logistic regression models comprise variables with *p* < 0.20 in bivariate analysis, and the results are presented as adjusted odds ratios (AORs) with 95% confidence intervals. The Hosmer–Lemeshow test was used to evaluate the model fit. The statistical significance was set at *p* < 0.05 (two-tailed).

## 3. Results

### 3.1. Baseline Characteristics of the Study Population

The characteristics of the 325 women who participated in the study are summed up in Table 1. The median age of enrolled participants was 36 years (IQR: 28–45), with the 25–34 age group being the most common (33.85%). Approximately one-third of the participants (33.54%) had attained a tertiary education. Most participants (87.31%) were non-smokers, while 58.51% were current or past consumers of alcohol. Regarding marital status, 74.92% were single, while 18.21% were married. Slightly over two-thirds (60.99%) reported a sexual debut of 16 to 19 years of age. Nearly half (48.92%) had engaged sexually with one to three partners in their lifetime. Overall, 86.38% were gravida, with 44.89% reporting one to two pregnancies. About 66.25% were on contraceptives. Only about a third (36.65%) had used a condom during the last sexual intercourse. Depo-Provera was the most common contraceptive method (32.00%), followed by condoms (13.85%). The prevalence of HIV was 64.51%, and all were on antiretroviral treatment. Signs of STIs were observed in 24.15% of participants. While 50.62% self-reported having once experienced abnormal vaginal discharge, 48.46% experienced abnormal vaginal itchiness, and 21.08% experienced genital warts, blisters, or ulcers (Table 1).

### 3.2. Candida Species and Bacterial Vaginosis Prevalence According to Age Groups

The internal control (human β-globulin gene) failed for one sample during processing of the Seegene Allplex™ Candidiasis Assay and for another sample during processing of the Allplex™ Bacterial Vaginosis Plus Assay. These specimens remained invalid after they were repeated; therefore, a total of 324 have valid Seegene Allplex™ Candidiasis Assay and Allplex™ Bacterial Vaginosis Plus Assay results. A proportion of 15.74% tested positive for any of the detected *Candida* spp. Among these, the majority tested positive for *C. albicans* (74.51%). The remaining were positive for non-*Candida albicans* species, primarily *C. glabrata* (21.57%), with *C. parapsilosis* (3.92%) and *C. krusei* (1.96%) appearing in minor occurrences. Co-occurrence of *Candida* species was not common; one case presented with *C. krusei* and *C. albicans*. The highest prevalence of *Candida* spp. was observed in the 18–29 age group (23.60%), with a significant downward trend in older age groups (R^2^ = 0.913; *p* = 0.015; Table 2).

A total of 52.85% tested positive for BV. A further 32.10% were BV-negative with vaginal microflora lacking appreciable *Lactobacillus* spp., 14.81% were BV-negative with *Lactobacillus*-rich microflora, and 1.23% were BV-intermediate. The prevalence of BV positivity was also highest in the 18–29 years age group (85.39%) and decreased significantly in older age groups (R^2^ = 0.931; *p* = 0.003). Conversely, the trends show a significant increase in BV negativity with age, from the 18–29-year-old age group (46.91%) to the 50–60-year-old age group (65.31%; R^2^ = 0.899, *p* = 0.003). Within BV-negative cases, microflora lacking *Lactobacillus* showed a significant upward trend with age (R^2^ = 0.899; *p* = 0.003), while *Lactobacillus*-rich microflora retained a steady prevalence across all age groups with no significant difference (R^2^ = 0.004; *p* = 0.822, Table 2). *Candida* and BV co-occurrence with an overall prevalence of 7.10% decreased significantly with age (R^2^ = 0.903, *p* = 0.041).

### 3.3. Distribution of Vaginal Pathogens and Protective Lactobacillus

Table 3 shows the distribution of bacterial species associated with the vaginal microbiome. *G. vaginalis* was the most prevalent species overall (77.47%), with the highest prevalence in women aged 18–29 years (85.39%) and declining steadily with age (R^2^ = 0.997, *p* < 0.001). *A. vaginae* (overall 57.10%) showed a similar pattern, peaking at 65.17% in the youngest group and decreasing across older age groups (R^2^ = 0.996, *p* < 0.001). BV-associated bacteria 2 was most common in the 18–29-year-old age group (51.69%), fluctuated in the 30–39 age group (38.83%) and the 40–49 age group (41.46%), and reached its lowest point in the 50–60 age group (30.61%) (R^2^ = 0.987, *p* = 0.001).

*Megasphaera* type 1 had an overall prevalence of 37.35%, peaking in the 18–29 age group (42.70%) and decreasing steadily to nearly half of the initial prevalence in the 50–60 age group (26.53%) (R^2^ = 0.990, *p* = 0.001). *Mobiluncus* species (overall 29.32%) was also most prevalent in the youngest group (32.58%) and remained consistent across the 30–39 age group, reaching its lowest prevalence of 20.41% in the 50–60 age group (R^2^ = 0.992, *p* = 0.000). Conversely, the *Lactobacillus* species (overall 22.84%) was lowest in the 30–39 group (16.50%), increased to 29.63% in the 40–49 group, and remained relatively higher in the 50–60 group (26.53%) compared with younger age groups (R^2^ = 0.971, *p* = 0.002, R^2^ = 0.971, *p* = 0.002). *B. fragilis* was rare overall (2.78%), absent in the oldest group, and showed a significant declining trend with age (R^2^ = 0.916, *p* = 0.011, Table 3).

As shown in Table 4, *G. vaginalis* was significantly more prevalent in BV-positive cases (95.24%) compared to BV-negative cases (57.24%; *p* < 0.001). *A.vaginae* was also more frequent in BV-positive women (83.93%) than in BV-negative women (27.63%; *p* < 0.001). BV-associated bacteria 2 was detected in 69.05% of BV-positive cases but only 7.24% of BV-negative cases (*p* < 0.001). Similarly, *Megasphaera* type 1 was identified in 69.05% of BV-positive cases, whereas it was nearly absent in BV-negative women (3.29%; *p* < 0.001). *Mobiluncus* spp. was present in 45.83% of BV-positive cases compared to 10.53% of BV-negative cases (*p* < 0.001). In contrast, *Lactobacillus* spp. was most prevalent in BV-negative cases (46.71%) but completely absent in BV-positive cases (*p* < 0.001). *B. fragilis* was the least prevalent overall, with 4.61% prevalence in BV-negative and 1.19% in BV-positive cases (*p* = 0.091).

Further stratification of BV-negative women revealed striking differences depending on *Lactobacillus* abundance. BV-negative cases with appreciable numbers of *Lactobacillus* spp. showed 100.00% prevalence of *Lactobacillus* spp., compared to only 22.12% in BV-negative cases lacking appreciable numbers of *Lactobacillus* spp. (*p* < 0.001). Conversely, the prevalence of *G. vaginalis* in BV-negative cases lacking appreciable numbers of *Lactobacillus* spp. nearly doubled that in BV-negative cases with appreciable numbers of *Lactobacillus* spp. (67.31% vs. 35.42%; *p* < 0.001). A similar case was noted in *A. vaginae*; however, the difference was borderline significant (16.67% vs. 32.69%; *p* = 0.051; Table 4).

**Figure 1:**
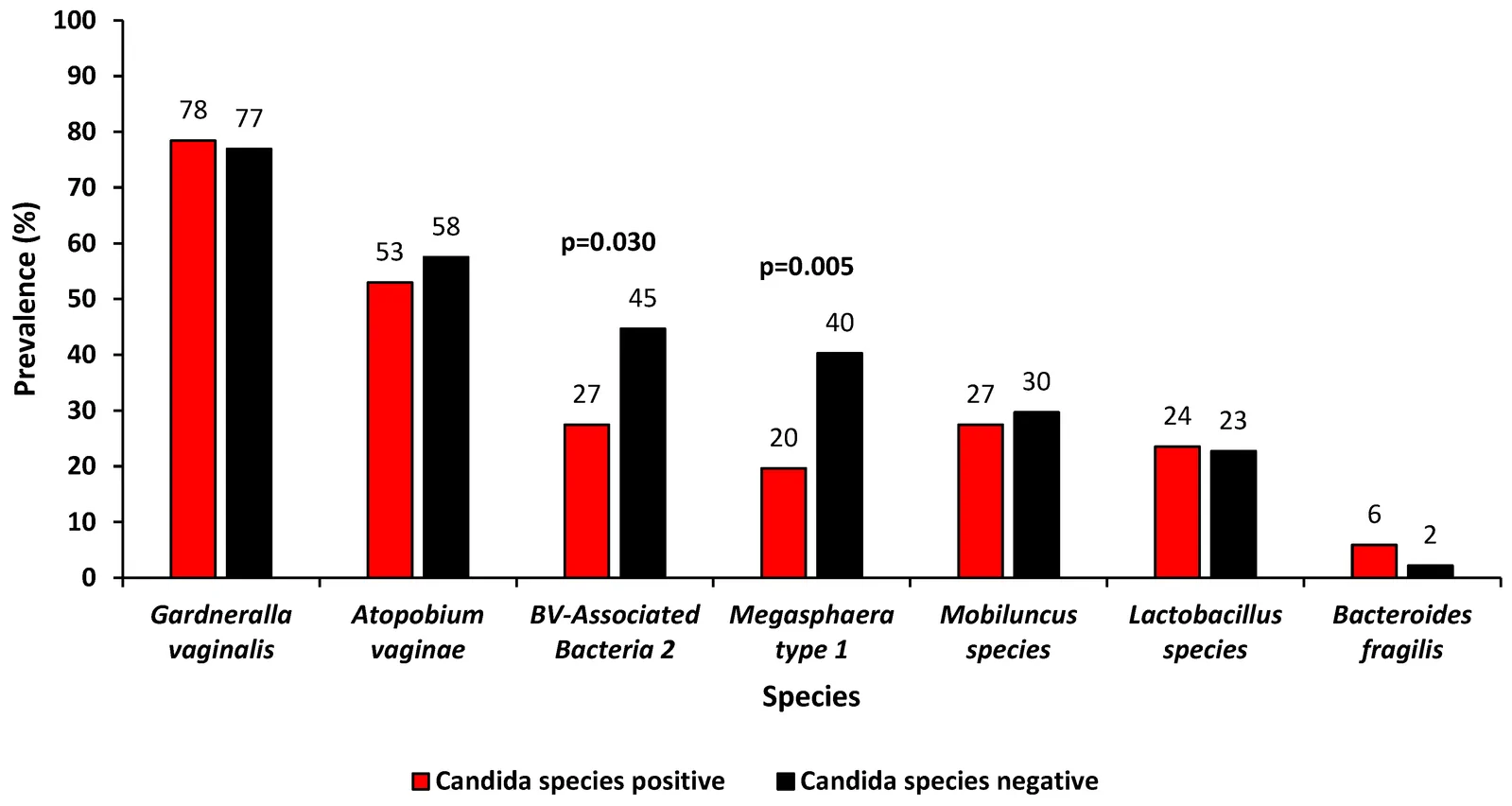
Distribution of vaginal dysbiosis-causing and protective bacteria according to *Candida* species status among women of the Eastern Cape Province.

The *Megasphaera* type 1 was significantly less prevalent among *Candida*-positive women (19.61%) compared to *Candida*-negative women (40.29%; *p* = 0.005), and similarly with BV-associated bacteria (27.45% compared with 44.69%, respectively; *p* = 0.030). *G. vaginalis* and *A. vaginae* were the most common species in both *Candida*-positive and *Candida*-negative women and did not differ between groups. Notably, *Lactobacillus* spp. prevalence was similar between groups (23.53% vs. 22.71%), indicating that the protective flora did not differ significantly by *Candida* status in this dataset. *Mobiluncus* species were also similar between the two groups. *B. fragilis* remained rare overall, with minimal contribution to group differences (Figure 1).

### 3.4. HIV-Stratified Candida Species and BV Prevalence

Among the 15.74% of individuals who tested positive for *Candida* spp., the distribution of detected *Candida* spp. was determined according to HIV status. *C. albicans* was the most common species among both HIV-negative (11.30%) and HIV-positive (11.96%) individuals. *C. glabrata* was more prevalent among the HIV-negative women (4.35%) than the HIV-positive women (2.87%). *C. parapsilosis* and *C. krusei* were found exclusively in HIV-positive individuals at 0.96% and 0.48%, respectively. The prevalence of *Candida* species and bacterial vaginosis, as well as their co-occurrence, was not statistically different between the HIV-positive and HIV-negative women (Table 5).

### 3.5. Factors Associated with Candida Species

In the bivariate analysis in Table 6, women presenting with STI signs were associated with increased odds of testing positive for *Candida* species (OR: 1.92; 95% CI: 1.01–3.65; *p* = 0.047), as were those with four to six lifetime sexual partners compared to those with one to three partners (OR: 2.41; 95% CI:1.26–4.63; *p* = 0.008). A sexual debut of 20–24 years was protective, with lower odds for being positive with *Candida* spp. compared to a debut at ≤15 years (OR: 0.27; 95% CI:0.09–0.84; *p* = 0.024). Similar observations were noted among women aged 50–60 years compared to those aged 18–29 years (OR: 0.29; 95% CI: 0.09–0.89; *p* = 0.031; Table 6). In a multivariate analysis with a perfect fit (Hosmer–Lemeshow test, *p* = 0.525) that was adjusted for age, concerning the factors such as presenting with STI signs, sexual debut, number of life-time sexual partners, and having been pregnant, having had a high number (four to six) of lifetime sexual partners was an independent predictor of *Candida* species positivity (AOR: 2.42, 95% CI: 1.21–4.82, *p* = 0.012). Other variables were not statistically significant after adjustment.

### 3.6. Factors Associated with Candida Species and BV Co-Occurrence

The investigation of factors associated with the co-occurrence of *Candida* spp. and BV was limited to women who were both *Candida* spp. and BV-positive and those who were negative for both. In crude models, women aged 40–49 years had lower odds of co-occurrence of *Candida* spp. and BV compared with those aged 18–29 years (OR: 0.24; 95% CI: 0.07–0.84; *p* = 0.025). A sexual debut between 20 and 24 years was associated with lower odds of co-occurrence of *Candida* spp. and BV compared to a sexual debut of ≤15 years (OR: 0.18; 95% CI: 0.04–0.78; *p* = 0.048). In contrast, women presenting with signs of STIs upon examination were associated with higher odds of *Candida* spp. and BV co-occurrences (OR: 4.50; 95% CI: 1.77–11.43; *p* = 0.002). After multivariate analysis, this association remained statistically significant (AOR: 4.66; 95% CI: 1.29–16.87; *p* = 0.019). Similarly, being HPV-positive was associated with increased odds of *Candida* spp. and BV co-occurrence (OR: 5.49; 95% CI: 1.55–19.43; *p* = 0.008); these associations remained in multivariate analysis (AOR: 7.28; 95% CI: 1.17–45.26; *p* = 0.033). Women who reported having had four to six lifetime sexual partners were also associated with increased odds of *Candida* spp. and BV co-occurrence than those who had one to three (OR: 4.42; 95% CI: 1.68–12.09; *p* = 0.004); the association remained significant after the multivariate analysis (AOR: 5.58; 95% CI: 1.16–26.82; *p* = 0.032, Table 7). These findings indicate that presenting with signs of STIs, HPV infection, and having had multiple sexual partners are independent predictors of *Candida* spp. and BV co-occurrence. However, those reporting a history of abnormal vaginal discharge or itchiness (OR: 2.32; 95% CI: 1.04–6.71; *p* = 0.040), alcohol consumption (OR: 2.98; 95% CI: 1.10–8.05; *p* = 0.032), and oral contraception use (OR: 6.53; 95% CI: 1.37–31.23; *p* = 0.019) in a bivariate analysis were associated with increased odds of *Candida* spp. and BV co-occurrences; after multivariate analysis, these associations were no longer statistically significant (Table 7).

## 4. Discussion

The surveillance of STIs in South Africa remains a crucial pursuit for safeguarding the health of the population [12]. Recent evidence indicates that effective STI screening and treatment may decrease the incidence of vertical HIV transmission and improve pregnancy outcomes [13]. Thus, the current study sought to investigate the association between *Candida* colonisation and BV, their co-occurrence, and the associated factors among women in the King Sabata Dalindyebo municipality. The findings demonstrate the prevalence of BV and *Candida* species: 52.85% and 15.74%, respectively. *C. albicans* was the most prevalent species, followed by *C. glabrata*, *C. parapsilosis*, and *C. krusei* as the least prevalent among rural and peri-urban women in the Eastern Cape. Of which, globally, *C. albicans* remains the most frequently isolated *Candida* species in VVC cases, followed by *C. glabrata* [14,15,16,17].

In the current findings, *Lactobacillus* spp. was more prevalent in BV-negative cases than in BV-positive cases. BV-positive women exhibited significant dominance of *G. vaginalis*, *A. vaginae*, BV-associated bacteria 2, *Megasphaera* type, and *Mobiluncus* species, whereas *Lactobacillus* spp. was nearly absent. Women aged 18–29 consistently had the highest prevalence of these species, with a relatively lower prevalence of *Lactobacillus* spp. Previous findings from South Africa show that younger reproductive-age women have increased microbial instability and repeated BV episodes, frequently caused by *G. vaginalis* and *A. vaginae* biofilm co-dominance [18,19]. The possible vulnerability of younger women in the current population is further highlighted by the high prevalence of *Megasphaera* type 1 (42.70% in 18–29 years) and *Mobiluncus* spp. (32.58% in 18–29 years), which have been linked with anaerobes to symptomatic BV and increased STI susceptibility, including HIV acquisition risk [20,21]. The current study also demonstrates a high prevalence of HIV at 64.51%. This might also highlight that the current population has increased susceptibility to STIs, as observed in similar high-burden contexts [22,23].

*Lactobacillus* depletion is most noticeable in the early reproductive years and gradually replenishes in later stages, though it rarely reaches the protective community state type I (CST-I), which is dominated by *Lactobacillus crispatus* [3,24]. Slightly more than half of the participants in the current study tested positive for BV and lacked normal vaginal microflora, including *Lactobacillus* species. Previous studies have associated BV with vaginal discharge syndrome [25], microbial imbalance, and susceptibility to HPV infection, dysplasia, and developing cervical cancer [26,27,28].

Almost half of the participants were categorised as BV-negative, and a large proportion had no detectable levels of *Lactobacillus* species. The prevalence of *Lactobacillus*-rich microflora remained constantly low across all age groups. An age-related decline in BV positivity was observed, with younger individuals (18–29 years) showing a higher prevalence of BV. Although a lack of *Lactobacillus* spp. is associated with adverse vaginal infection, it is also associated with a shift to a new, non-pathogenic microbial state that nevertheless lacks the conventional indicators of ideal vaginal health rather than a recovery of *Lactobacillus* species [29]. Pathological dysbiosis in the vaginal microbiota is characterised by a decline in *Lactobacillus* spp. dominance, increased microbial diversity, and visible clinical signs [30].

The current findings demonstrate an inverse relationship between age and *Candida* prevalence, the youngest group showing the highest prevalence. A decline in *Candida* positivity with increasing age was observed. The current findings revealed that women aged 40–49 were less likely to test positive for *Candida* species. Those in the 50–60 age group were less likely to present with co-infections of *Candida* and BV than women aged 18–29. This may be attributed to behavioural factors, which may be more common in younger populations. These may include higher numbers of sexual partners or specific contraceptive choices that may play a primary role in the overgrowth of *Candida* [31,32].

A significant association was observed between sexual history and testing positive for *Candida* species. Having had four to six lifetime sexual partners was an independent predictor of testing positive for *Candida* species compared with those who had fewer partners. Furthermore, a later sexual debut (20–24 years) appeared to be protective against *Candida* compared with those whose sexual debut occurred at 15 years or younger, although this association was not retained after adjustment. In previous studies, an early sexual debut has been identified as a risk factor for STIs [33]. This observation is also stated by earlier studies in sub-Saharan Africa and South Africa, which demonstrated that adolescents initiating sexual activity before age 16 had significantly higher rates of HIV and other STIs [34,35]. This was recently confirmed by a multi-country analysis, showing that adolescents initiating sex before age 16 had significantly higher odds of new HIV infections compared with those who had a delayed debut [33]. Increased sexual exposure may contribute to the overgrowth of *Candida* [31] and general instability of the vaginal microflora [36]. However, no association was found for the conventional clinical markers, such as HIV status and the use of hormonal contraceptives even though the previous studies have linked the immunosuppressive effect of HIV to susceptibility to *Candida* species and VVC [37,38,39]. This suggests that behavioural history may be a more sensitive indicator for susceptibility to *Candida* in the current population than endocrine or systemic immunological factors [31,40]. Previous studies emphasised that behavioural exposures, such as hygiene and lifestyle, contribute to STI symptoms and microbial instability [33].

In the current findings, those with signs of STIs had increased odds of testing positive for *Candida* and BV simultaneously, despite possible microbial competition driven by the dominance of pathogens such as *Trichomonas vaginalis*, *Neisseria gonorrhoeae*, and *Chlamydia trachomatis*, which often alter vaginal pH or trigger an immune response hostile to the colonisation of *Candida* and BV-associated anaerobic bacteria. This antagonistic competition may effectively suppress *Candida* overgrowth and the establishment of BV biofilms, such that individuals with symptomatic STIs may present with no co-occurrence of *Candida* and BV [3,15,41].

Testing positive for HPV was an independent predictor of *Candida*–BV co-occurrence, underscoring possible local immune–microbial interactions and instability within the vaginal microbiome. This may be explained by several mechanisms. *Candida albicans*, the dominant vaginal fungal species, possesses a morphological plasticity that enables the transition from commensal blastoconidia to invasive hyphae [2,3,15]. Hyphal forms express virulence factors, such as candidalysin, which disrupt epithelial integrity and trigger the inflammatory pathway [2,20]. Vaginal epithelial recognition of hyphae activates the innate c-Fos/MAPK signalling pathway, leading to neutrophil recruitment and resulting in symptomatic inflammation. On the other hand, BV-associated bacteria (*G. vaginalis*, *A. vaginae*, *Megasphaera*) promote epithelial disruption and mucosal instability, creating a permissive environment for fungal overgrowth [42,43,44]. The protective role of *Lactobacillus* spp. is diminished, as the loss of lactic acid and hydrogen peroxide production reduces colonisation resistance [3,4,5]. The abundance of lactic acid and hydrogen peroxide-producing *Lactobacillus* maintains vaginal microbial homeostasis by maintaining a lower acidic pH (pH ~3.5–4.5), resisting bacterial and fungal pathogenicity, and reducing the risk of viral infection, including susceptibility to sexually transmitted pathogens [2,3]. The disruption of this acidic milieu through reduced *Lactobacillus* density facilitates the overgrowth of anaerobic bacteria and enhances the likelihood of fungal proliferation and viral persistence [2].

HPV infection itself is known to shift the microbiome toward BV-associated taxa, facilitating viral persistence and cervical lesion progression [28,29,30]. Previous studies have consistently reported that HPV infection is accompanied by shifts toward a BV-associated microbiome, particularly *G. vaginalis*, *A. vaginae*, and *Megasphaera* species, which promote viral persistence and progression to cervical lesions [42,43,44]. The co-occurrence of *Candida* and BV in HPV-positive women further underscores the role of epithelial disruption and mucosal inflammation in facilitating fungal overgrowth [20,44].

In line with previous surveillance in South Africa [45], syndromic STI presentations frequently coincide with vaginal dysbiosis; participants exhibiting signs of STIs had increased odds of *Candida* positivity and decreased *Lactobacillus* dominance. In a crude analysis, oral contraceptive use was also linked to higher odds of co-infection, which is consistent with previous studies associating hormonal contraceptives with changes in the microbiota and cytokine profiles in the vagina, making it more susceptible to *Candida* and BV [22,46,47].

Several limitations are notable in this study. This is a cross-sectional study, in which observations and conclusions were drawn at a single point in time. Thus, the study does not establish cause-and-effect relationships between the behavioural and clinical factors attributed and the observed factor attributed to dysbiosis. This study only highlights the associations between these variables. This study was conducted in a specific region and population (at Mthatha Gateway Clinic in the KSD Municipality, Eastern Cape Province). The population was also excluded from those who were menstruating or pregnant, which limits the generalisability of the findings to other populations and regions of the Eastern Cape and South Africa. Using samples collected from the cervix rather than the vagina might limit the detection of *Candida* species, as the vagina is commonly considered their primary colonisation site. The method of data collection included a questionnaire; participants were asked to recall past events, such as sexual behaviour, habits related to alcohol and smoking, hygienic practices, history, and signs related to STIs and vaginal infection. Self-reported data from participants can be subject to recall bias or social desirability bias. Memories can be inaccurate, and participants may struggle to recall certain events. It is also important to note that in many societies and cultures, including those of the Eastern Cape, sexually related activities and events are regarded as private and sensitive subjects [48]. A small sample size, particularly among women with *Candida*–BV co-occurrence, also served as a study limitation. Consequently, some odds ratios were accompanied by wide confidence intervals, reflecting reduced precision despite statistically significant associations. These findings should therefore be interpreted with caution and confirmed in larger, adequately powered prospective studies. It is therefore recommended that further studies on this subject adopt a longitudinal study design and include diverse populations to better understand the associations and improve generalisability. In addition, it is important to conduct molecular analyses and tests for more microbes related to STIs and vaginal infections, as well as to further explore the intricate complexities involved in vaginal dysbiosis.

## 5. Conclusions

This study demonstrated a substantial burden of bacterial vaginosis, *Candida* species, and their co-occurrence among women in a predominantly HIV-positive population, with the highest prevalence observed among women aged 18–29 years. Multiple lifetime sexual partners were independently associated with *Candida* species positivity, while current STI symptoms, HPV infection, and having four to six lifetime sexual partners significantly increased the likelihood of *Candida*–BV co-occurrence. These findings suggest that behavioural and biological risk factors can jointly contribute to vaginal dysbiosis and mixed genital tract infections. Longitudinal studies are warranted to clarify the temporal relationships between HPV infection, vaginal dysbiosis, and mixed genital tract infections. Data generated from this study could inform targeted public health interventions.

## Figures and Tables

**Table 1:** The characteristics of study participants.

Characteristics	All Participants	
	*n*	%
Age (years)		
18–29	89	27.47
30–39	103	31.79
40–49	84	25.62
50–60	49	15.12
Level of Education		
Primary (Grade 0–7)	21	6.48
Secondary (Grade 8–12)	194	59.88
Tertiary	109	33.64
Marital status ^Σ^		
Single	242	74.69
Married/cohabiting	65	20.06
Widowed/divorced	17	5.25
Ever smoke ^Σ^		
Yes	41	12.69
No	282	87.31
Sexual debut, years; Median (IQR)	18 (17−20)	
≤15 years	37	11.42
16–19 years	197	60.80
20–24 years	74	22.84
25–34 years	16	4.94
Number of lifetime sexual partners; median (IQR) ^Σ^	4 (3−5)	
1–3	158	48.92
4–6	129	39.94
≥7	36	11.15
Number of sexual partners past 3 months; median (IQR) ^Σ^	1 (1−1)	
Frequency of vaginal sexual intercourse in the past 1 month; Median (IQR) ^Ψ^	1 (0−1)	
Ever consumed alcohol ^Σ^		
No	135	41.80
Yes	189	58.51
Ever pregnant ^Σ^		
No	44	13.63
Yes	279	86.38
Currently on contraception ^Σ^		
No	109	33.75
Yes	214	66.25
Method of contraception		
None	109	33.54
Oral Contraceptives	18	5.54
Condom	45	13.85
Mirena, ParaGard, Implanon or Nexplanon	14	4.31
Depo-Provera	104	32.00
Nur-Isterate	17	5.23
Withdraw	13	4.00
Injection, but not sure which one	5	1.54
Condom use during last intercourse ^Σ^		
No	204	63.35
Yes	118	36.65
Presenting with signs of STI		
No	246	76.16
Yes	78	24.15
Abnormal Vaginal discharge		
No	160	49.38
Yes	164	50.62
Vaginal itchiness		
No	167	51.54
Yes	157	48.46
Genital warts, blisters, or ulcers		
No	255	78.95
Yes	68	21.05
HIV Status ^Σ^		
Negative	115	35.49
Positive	209	64.51

^Σ^ Some participants are non-responders in this section. Therefore, the total number of participants decreased from the original 325. ^Ψ^ There are two non-responders among those who were once pregnant.

**Table 2:** Prevalence of *Candida* species and bacterial vaginosis among women of the Eastern Cape Province.

Species	Overall	18–29 Years	30–39 Years	40–49 Years	50–60 Years		
	% (*n*/324)	% (*n*/89)	% (*n*/103)	% (*n*/82)	% (*n*/49)	R^2^	*p* for Trend
*Candida* spp.	15.74 (51)	23.60 (21)	14.56 (15)	13.41 (11)	8.16 (4)	0.913	**0.015**
BV-negative	46.91 (152)	39.33 (35)	41.75 (43)	50.00 (41)	65.31 (32)	0.899	**0.003**
BV-negative ^¥^	14.81 (48)	16.85 (15)	9.71 (10)	19.51 (16)	14.29 (7)	0.004	0.822
BV-negative ^§^	32.10 (104)	22.47 (20)	32.04 (33)	30.49 (25)	51.02 (25)	0.899	**0.003**
BV-intermediate	1.23 (4)	1.12 (1)	1.94 (2)	1.22 (1)	0.00 (0)	…	…
BV-positive	51.85 (168)	85.39 (53)	56.31 (58)	47.56 (39)	34.69 (17)	0.931	**0.003**
*Candida* and BV-positive	7.10 (23)	14.61 (13)	5.83 (6)	4.88 (4)	0.00 (0)	0.903	**0.041**

One participant had no age data. A bold *p*-value indicates significance. ^¥^ Normal vaginal microflora, with appreciable numbers of *Lactobacillus* spp. ^§^ Normal vaginal microflora but lacking appreciable numbers of *Lactobacillus* spp.

**Table 3:** Vaginal microbial flora distribution across different age groups among women in the Eastern Cape Province.

Species	Overall	18–29 Years	30–39 Years	40–49 Years	50–60 Years		
	% (*n*/324)	% (*n*/89)	% (*n*/103)	% (*n*/82)	% (*n*/49)	R^2^	*p* for Trend
*Gardnerella vaginalis*	77.47 (251)	85.39 (79)	76.70 (79)	76.83 (63)	65.31 (32)	0.997	**<0.001**
*Atopobium vaginae*	57.10 (185)	65.17 (58)	54.37 (56)	53.66 (44)	53.06 (26)	0.996	**<0.001**
*BV*-Associated Bacteria 2	41.98 (135)	51.69 (46)	38.83 (40)	41.46 (34)	30.61 (15)	0.987	**0.001**
*Megasphaera* type 1	37.35 (121)	42.70 (38)	40.78 (42)	32.93 (27)	26.53 (13)	0.990	**0.001**
*Mobiluncus* species	29.32 (95)	32.58 (29)	32.04 (33)	28.05 (23)	20.41 (10)	0.992	**<0.001**
*Lactobacillus* species	22.84 (74)	22.47 (20)	16.50 (17)	29.27 (24)	26.53 (13)	0.971	**0.002**
*Bacteroides fragilis*	2.78 (9)	2.25 (2)	3.88 (4)	3.66 (3)	0.00 (0)	0.916	**0.011**

One participant had no age data. A bold *p*-value indicates significance.

**Table 4:** Distribution of vaginal dysbiosis-causing and protective bacteria according to bacterial vaginosis status.

Species	BV-Negative	BV-Positive	
	% (*n*/152)	% (*n*/168)	*p*-Value
*Gardnerella vaginalis*	57.24 (87)	95.24 (160)	**<0.001**
*Atopobium vaginae*	27.63 (42)	83.93 (141)	**<0.001**
BV-Associated Bacteria 2	7.24 (11)	74.40 (125)	**<0.001**
*Megasphaera* type 1	3.29 (5)	69.05 (116)	**<0.001**
*Mobiluncus* species	10.53 (16)	45.83 (77)	**<0.001**
*Lactobacillus* species	42.76 (65)	2.98 (5)	**<0.001**
*Bacteroides fragilis*	4.61 (7)	1.19 (2)	0.091
**Species**	**BV-Negative** **^¥^**	**BV-Negative** **^§^**	
	**% (*n*/48)**	**% (*n*/104)**	***p*-Value**
*Gardnerella vaginalis*	35.42 (17)	67.31 (70)	**0.000**
*Atopobium vaginae*	16.67 (8)	32.69 (34)	0.051
BV-Associated Bacteria 2	2.08 (1)	9.62 (10)	0.175
*Megasphaera* type 1	2.08 (1)	3.84 (4)	>0.999
*Mobiluncus* species	6.25 (3)	12.50 (13)	0.394
*Lactobacillus* species	100.00 (48)	16.35 (17)	**<0.001**
*Bacteroides fragilis*	2.08 (1)	5.77(6)	0.433

^¥^ Normal vaginal microflora, with appreciable numbers of *Lactobacillus* spp. ^§^ Normal vaginal microflora but lacking appreciable numbers of *Lactobacillus* spp. BV: Bacterial vaginosis. A bold *p*-value indicates significance.

**Table 5:** Prevalence of *Candida* species and bacterial vaginosis and their co-occurrence by HIV status.

	HIV-Negative		HIV-Positive		*p*-Value
	%	*n*/N	%	*n*/N	
*Candida* spp. negative	82.61	95/115	84.21	176/209	0.633
*Candida* spp. positive	17.39	20/115	14.86	31/209	
BV-positive	45.61	52/114	55.37	115/204	0.079
BV-negative	54.39	62/114	43.63	89/204	
*Candida*-negative and BV-positive	37.72	43/114	49.26	100/203	0.060
*Candida*-positive and BV-positive	7.89	9/114	6.90	14/203	0.822
*Candida*-positive and BV-negative	9.65	11/114	8.37	17/203	0.686
*Candida*-negative and BV-negative	44.74	51/114	35.47	72/203	0.119

One participant had an unknown HIV status. BV: Bacterial vaginosis.

**Table 6:** Factors associated with being positive for *Candida* species (bivariate analysis).

		*Candida* Species					
Characteristics	Total	Positive	%	Negative	%	OR (95% CI)	*p*-Value
	324	51	15.74	273	84.26	-	-
HIV Status ^Σ^							
Negative	115	20	17.39	95	82.61	Reference	
Positive	208	31	14.90	177	85.10	0.84 (0.45–1.55)	0.570
Age Range (years) ^Σ^							
18–29	89	21	23.60	68	76.40	Reference	
30–39	103	15	14.56	88	85.44	0.55 (0.27–1.15)	0.113
40–49	82	11	13.41	71	86.58	0.50 (0.23–1.12)	0.092
50–60	49	4	8.16	45	91.84	0.29 (0.09–0.89)	**0.031**
Bacterial Vaginosis ^&^							
Normal ^¥^	48	8	16.67	40	83.33	Reference	
Normal ^§^	104	20	19.23	84	80.77	1.19 (0.48–2.94)	0.705
Positive	167	23	13.77	144	86.23	0.80 (0.33–1.92)	0.615
Intermediate	4	0	0.00	4	100.00	…	
Presenting with STI							
No	246	33	13.41	211	85.77	Reference	
Yes	78	18	23.08	60	76.92	1.92 (1.01–3.65)	**0.047**
Abnormal Vaginal discharge							
No	160	24	15.00	135	84.38	Reference	
Yes	164	27	16.46	136	82.93	1.12 (0.61–2.03)	0.718
Vaginal itchiness							
No	167	24	14.37	142	85.03	Reference	
Yes	157	27	17.20	129	82.17	1.24 (0.68–2.26)	0.484
Genital warts, blisters, or ulcers ^Σ^							
No	255	41	16.08	213	83.53	Reference	
Yes	68	10	14.71	57	83.82	0.91 (0.43–1.93)	0.809
Sexual debut ^Σ^							
≤15 years	37	9	24.32	28	75.68	Reference	
16–19 years	195	32	16.24	163	82.74	0.61 (0.26–1.42)	0.251
20–24 years	74	6	8.11	68	91.89	0.27 (0.09–0.84)	**0.024**
25–34 years	16	4	25.00	12	75.00	1.04 (0.27–4.03)	0.958
Number of lifetime sexual partners ^Σ^							
1–3	157	17	10.83	140	89.17	Reference	
4–6	128	29	22.66	99	77.34	2.41 (1.26–4.63)	**0.008**
≥7	36	5	13.89	31	86.11	1.33 (0.46–3.87)	0.603
Ever pregnant ^Σ^							
No	45	11	24.44	34	75.56	Reference	
Yes	277	40	14.44	237	85.56	0.51 (0.24–1.08)	0.079
Contraception ^Σ^							
None	109	19	17.43	90	82.57	Reference	
Oral Contraceptives	18	5	27.78	13	72.22	1.82 (0.58–5.72)	0.304
Condom	44	7	15.91	37	84.09	0.90 (0.35–2.31)	0.821
Mirena, ParaGard, Implanon or Nexplanon	14	1	7.14	13	92.86	0.36 (0.05–3.00)	0.345
Depo-Provera	103	12	11.65	91	88.35	0.63 (0.29–1.36)	0.237
Nur-Isterate	16	5	31.25	11	68.75	2.15 (0.67–6.92)	0.198
Withdraw	13	2	15.38	11	84.62	0.86 (0.18–4.21)	0.854
Injection, but not sure which one	4	0	0.00	4	100.00	…	…

One specimen had an invalid internal control for *Candida* results; therefore, the total is 324 instead of 325. ^Σ^ Some participants are non-responders in this section. ^&^ Bacterial vaginosis was identified by quantitative detection of *Lactobacillus* spp., *Gardnerella vaginalis*, *Atopobium vaginae*, *Bacteroides fragilis*, *Mobiluncus* spp., *Megasphaera* type 1, and BV-associated bacteria 2 (BVAB2) using a multiplex real-time PCR assay. ^¥^ Normal vaginal microflora, with appreciable numbers of *Lactobacillus* spp. ^§^ Normal vaginal microflora but lacking appreciable numbers of *Lactobacillus* spp. A bold *p*-value indicates significance.

**Table 7:** Factors associated with the co-occurrence of *Candida* species and bacterial vaginosis (BV).

Characteristics	N	*Candida*–BV-Positive % (*n*)	*Candida*–BV-Negative % (*n*)	OR (95% CI)	*p*-Value	AOR (95% CI)	*p*-Value
	147	15.63 (23)	84.35 (124)				
Age group (years)							
18–29	40	32.50 (13)	67.50 (27)	Reference		Reference	
30–39	40	15.00 (6)	85.00 (34)	0.37 (0.12–1.09)	0.071	0.56 (0.13–2.46)	0.446
40–49	38	10.53 (4)	89.47 (34)	0.24 (0.07–0.84)	**0.025**	1.11 (0.17–7.45)	0.915
50–60	28	0.00 (0)	100.00 (28)	…	**…**		
Marital status ^Σ^							
Single	100	20.00 (20)	80.77 (80)	Reference			
Married/Cohabiting	36	8.33 (3)	92.68 (33)	0.36 (0.10–1.31)	0.121		
Divorced/Widowed	10	0.00 (0)	100.00 (10)	…	…		
Education level ^Σ^							
Primary (Grade 0–7)	12	0.00 (0)	100.00 (12)	…			
Secondary (Grade 8–12)	93	12.90 (12)	87.10 (81)	0.40 (0.16–1.01)	0.053		
Tertiary	41	26.83 (11)	73.17 (30)	Reference			
HIV status ^Σ^							
Negative	60	15.00 (9)	85.00 (51)	Reference			
Positive	86	16.27 (14)	83.72 (72)	1.10 (0.44–2.74)	0.835		
Smoker ^Σ^							
No	129	13.95 (18)	86.05 (111)	Reference		Reference	
Yes	17	29.41 (5)	70.59 (12)	2.57 (0.81–8.16)	0.110	1.80 (0.29–11.36)	0.531
Ever Consumed Alcohol ^Σ^							
No	69	8.70 (6)	91.30 (63)	Reference			
Current/past	77	22.08 (17)	77.92 (60)	2.98 (1.10–8.05)	**0.032**	0.78 (0.20–3.06)	0.780
Shower after sex ^Σ^							
Yes, almost immediately	33	9.09 (3)	90.91 (30)	Reference			
Not immediately, could be hours after	102	17.65 (18)	82.35 (84)	2.14 (0.59–7.80)	0.247		
Sometimes	11	18.18 (2)	81.82 (9)	2.22 (0.32–15.43)	0.419		
Alcohol/drugs during sex ^Σ^							
No	132	14.71 (20)	85.29 (112)	Reference			
Yes	14	21.43 (3)	78.57 (11)	1.54 (0.40–6.02)	0.534		
Always use a condom ^Σ^							
No/Sometimes	94	12.77 (12)	87.23 (82)	Reference			
Yes	52	21.15 (11)	78.85 (41)	1.83 (0.75–4.51)	0.187		
Contraception ^Σ^							
None	55	10.91 (6)	89.09 (49)	Reference		Reference	
Oral Contraceptives	9	44.44 (4)	55.56 (5)	6.53 (1.37–31.23)	**0.019**	4.78 (0.53–43.13)	0.163
Condom	22	27.27 (6)	72.73 (16)	3.06 (0.87–10.85)	0.083	0.44 (0.07–2.67)	0.369
Mirena, ParaGard, Implanon or Nexplanon	6	16.67 (1)	83.33 (5)	1.63 (0.16–16.43)	0.677	3.01 (0.09–97.19)	0.535
Depo-Provera	43	11.63 (5)	88.37 (38)	1.08 (0.31–3.79)	0.911	0.28 (0.05–1.49)	0.136
Nur-isterate	7	14.29 (1)	85.71 (6)	1.36 (0.14–13.31)	0.791	0.47 (0.03–7.52)	0.595
Withdraw	2	0.00 (0)	100.00 (2)	…	…		
Injection but not sure which one	2	0.00 (0)	100.00 (2)	…	…		
Ever pregnant ^Σ^							
No	20	30.00 (6)	70.00 (14)	Reference		Reference	
Yes	126	13.49 (17)	86.51 (109)	0.36 (0.12–1.08)	0.068	0.32 (0.05–2.08)	0.234
Presenting with STI ^Σ^							
No	110	10.00 (11)	90.00 (99)	Reference		Reference	
Yes	36	33.33 (12)	66.67 (24)	4.50 (1.77–11.43)	**0.002**	4.66 (1.29–16.87)	**0.019**
Abnormal Vaginal discharge or itching ^Σ^							
No	80	10.00 (8)	90.00 (72)	Reference		Reference	
Yes	66	22.73 (15)	77.27 (51)	2.65 (1.04–6.71)	**0.040**	0.97 (0.25–3.78)	0.963
Vaginal itchiness ^Σ^							
No	76	10.53 (8)	89.47 (68)	Reference		Reference	
Yes	70	21.43 (15)	78.57 (55)	2.32 (0.92–5.87)	0.076	1.40 (0.37–5.37)	0.622
Genital warts, blisters, or ulcers ^Σ^							
No	116	13.79 (16)	86.21 (100)	Reference			
Yes	30	23.33 (7)	76.67 (23)	1.90 (0.70–5.16)	0.206		
HPV Status							
Negative	59	5.08 (3)	94.92 (56)	Reference		Reference	
Positive	88	22.73 (20)	77.27 (68)	5.49 (1.55–19.43)	**0.008**	7.28 (1.17–45.26)	**0.033**
Sexual debut (years) ^Σ^							
≤15 years	14	28.57 (4)	71.43 (10)	Reference		Reference	
16–19 years	79	18.99 (15)	81.01 (64)	0.59 (0.16–2.13)	0.416	1.35 (0.20–9.16)	0.759
20–24 years	43	6.98 (3)	93.02 (40)	0.19 (0.04–0.98)	**0.047**	0.79 (0.08–7.45)	0.838
≥25 years	10	10.00 (1)	90.00 (9)	0.28 (0.03–0.297)	0.289	0.35 (0.02–710)	0.493
Number of lifetime sexual partners ^Σ^							
1–3	79	7.59 (6)	92.41 (73)	Reference		Reference	
4–6	57	26.32 (15)	73.68 (42)	4.35 (1.57–12.05)	**0.005**	5.58 (1.16–26.82)	**0.032**
≥7	9	22.22 (2)	77.78 (7)	3.48 (0.59–20.58)	0.170	1.74 (0.13–24.28)	0.679

^Σ^ Some participants are non-responders in this section. Therefore, the total number of participants decreased from the original 325. In a multivariate analysis, the Hosmer–Lemeshow test demonstrated a good fit (*p* = 0.264). The variables with adjusted odds ratios were the only ones included in multivariate analysis. A bold *p*-value indicates significance.

## Data Availability

The data are available upon request from the corresponding author.

## Abbreviations

The following abbreviations are used in this manuscript:

BV	Bacterial vaginosis
BVAB2	Bacterial vaginosis-associated bacteria 2
CI	Confidence Interval
HIV	Human Immunodeficiency Virus
HPV	Human papillomavirus
IQR	Interquartile range
KSD	King Sabata Dalindyebo
STIs	Sexually Transmitted Infections
OR	Odds Ratio
PCR	Polymerase chain reaction
UDG	Uracil-DNA glycosylase
VVC	Vulvovaginal candidiasis
